# shinyGISPA: A web application for characterizing phenotype by gene sets using multiple omics data combinations

**DOI:** 10.1371/journal.pone.0192563

**Published:** 2018-02-07

**Authors:** Bhakti Dwivedi, Jeanne Kowalski

**Affiliations:** 1 Winship Cancer Institute, Emory University, Atlanta, Georgia, United States of America; 2 Department of Biostatistics and Bioinformatics, Rollins School of Public Health, Atlanta, Georgia, United States of America; Wageningen UR Livestock Research, NETHERLANDS

## Abstract

While many methods exist for integrating multi-omics data or defining gene sets, there is no one single tool that defines gene sets based on merging of multiple omics data sets. We present shinyGISPA, an open-source application with a user-friendly web-based interface to define genes according to their similarity in several molecular changes that are driving a disease phenotype. This tool was developed to help facilitate the usability of a previously published method, Gene Integrated Set Profile Analysis (GISPA), among researchers with limited computer-programming skills. The GISPA method allows the identification of multiple gene sets that may play a role in the characterization, clinical application, or functional relevance of a disease phenotype. The tool provides an automated workflow that is highly scalable and adaptable to applications that go beyond genomic data merging analysis. It is available at http://shinygispa.winship.emory.edu/shinyGISPA/.

## Introduction

Identification of driver genes in a sample phenotype remains quintessential in cancer genomics research for which expression data has been typically analyzed to identify genes with significant differences between groups of similar phenotypes. The increasing availability of molecular data has shifted the focus away from examining changes in a single data type (e.g., expression) to examining changes using a combination of multiple data types (e.g., expression, methylation, copy number, and variant allele frequencies). The examination of multiple molecular changes has been referred to more generally as genomic data integration for which two approaches, data merging and results merging have been proposed [1]. A result merging approach is the simpler of the two and hence, the more popular. With this approach, genes are defined as statistically significant within each data type and then combined among data types by their intersection. The result is a list of genes that are commonly identified as significantly different among phenotypes resulting from several independent analyses of each data type and therefore thought to collectively explain substantial phenotypic changes. This approach suffers from the major limitation that, as the number of data types increases, the intersection becomes smaller and smaller and, hence, it is not scalable. Additionally, the merging of resulting gene sets by assessing each gene list from independently analyzed data types is not practical. Alternatively, a data merging approach combines the several different data types, and analysis is performed on the combined data set. Many data integration approaches [2], which are outside the scope of this paper, are limited in both the number and type of data permitted and are therefore not scalable, while others impose functional relationships among data types and are therefore not generalizable. We developed a data merging method [3] for genomic data integration, Gene Integrated Set Profile Analysis (GISPA) that is both scalable and generalizable. The usefulness of such a method is likely to go undetected in the absence of an available interactive computational tool. Here, we introduce a web-tool for our GISPA method to increase its usability and accessibility to a wider audience. The shinyGISPA tool is based on our GISPA method and is designed to characterize the molecular tumor profile of a single sample relative to other, comparison samples based on changes (increasing/decreasing) among several diverse, genome-wide data types. The tool has the following features: 1) it defines a set of genes by comparing them to all other genes in the user-input data set; 2) it is able to characterize a single sample’s molecular changes; and 3) it compares molecular changes among more than two phenotypic groups based on as few as a single sample per group. Most generally, the tool enables a user to perform genomic data integration by merging any number of molecular data types in any combination through a user-defined molecular profile that indicates direction (increased/decreased) of change within each data type. For example, a user may specify a profile of decreased gene expression with increased methylation and decreased copy change or increased gene expression with increased variant allele frequency and thus use a different number of data types in different combinations. The output is a list of gene sets that are ranked according to their level of support for a user-defined molecular profile that is a characteristic of a phenotype.

## Methods and implementation

**Fig 1:**
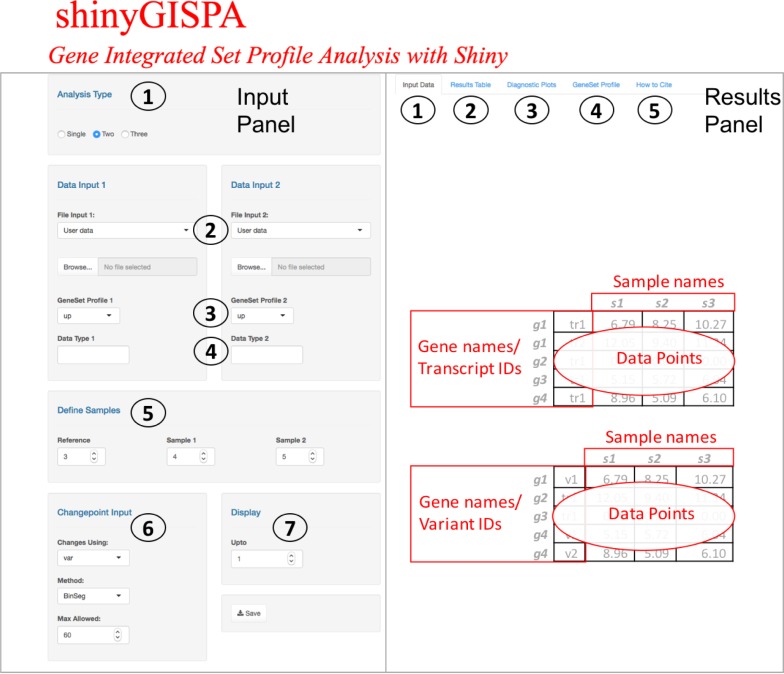
A screenshot of the shinyGISPA web user-interface for a two-feature analysis.

The shinyGISPA is an interactive web-based application, implemented using the Shiny R package [4]. The tool is hosted on a CentOS server running the Shiny Server program designed to host R Shiny applications. The source code is written in the R programming language (https://www.r-project.org/) and is freely available to download from GitHub (https://github.com/BhaktiDwivedi/shinyGISPA) and Bioconductor (http://bioconductor.org/packages/GISPA/). The main R packages used for data analysis and graphics were changepoint v2.2.2 [5] and HH v3.1–34 [6]. Fig 1 demonstrates the user-interface of shinyGISPA tool.

The shinyGISPA user-interface is comprised of two sections: the input and resultpanels as shown in Fig 1. The input table panel consists of seven input fields that a user can modify: (1) *Select the analysis type*. User selects a one-, two-, or three-feature analysis with the “Analysis Type” option. Here, feature is defined as a specific data type (e.g., expression, methylation, somatic mutation, and copy number variation). An example of one-feature analysis is identifying gene sets with expression changes, a two-feature analysis is based on a combination of any two data types, e.g., identifying gene sets that exhibit gene expression and copy change changes, while a three-feature analysis is based on a combination of any three data types, e.g., identifying gene sets that exhibit expression, copy number, and methylation changes. (2) *Upload the input data*: Select the file to be uploaded for each data type selected in (1) with choose file option under “File Input”. An example data set can also be uploaded to run the method. The input data can be genome-wide or a subset of data based on prior knowledge derived from one of biological processes, pathways, biomarkers discovery, or prior genomic analysis. The input data set must be a tab-delimited file format, where each row represents a gene (or a user-defined id) and each column a sample. As an analysis tool, the input data should be pre-processed and normalized within samples. When running shinyGISPA on multiple data types (two-feature or three-feature analysis), the first column of each input data set must correspond to a variable that is common to all input data sets. For instance, the input data sets should have common gene names, type, or IDs in the first column as these are used to merge the data tables into a single data set for analysis. The number of rows representing gene names, probes, variants, or any other id may or may not be the same. The tool requires a minimum of at least 10 genes and three samples to run. No duplicated rows or columns are allowed or else the analysis will be stopped. Rows with zero variance across all samples are excluded from the analysis. (3) *Select the gene set profile*. Select the “Gene Set Profile” button to define the desired direction of change in the gene set separately for each data type. Here, a user can select either “up” or “down” profile to define genes with increased or decreased changes in each data type. (4) *Specify the data type*. This option allows the users to label each uploaded data type, for instance, whether the data represents array-based expression, RNA-Seq expression, somatic mutation, copy number change, or methylation. (5) *Define samples*. User defines sample classes (or groups) in the uploaded data set by specifying the sample columns corresponding to the ‘Reference’ (sample of interest to characterize gene set profile on) and remaining two samples with which to compare the reference sample against. (6) *Select the change point input*. User may select the default settings or modify the change point detection method [5] parameters to find the optimal break points based on the profile statistic [3]. The type of changes in the data set may be based on using “mean”, “variance”, or “both” with user-specified method (“AMOC”, “BinSeg”, “PELT”, or “SeqNeigh”). (7) *Display*. Users can visualize the gene sets by change points with the “up to” button. At the bottom of the page, there is a “Save” button to download the “Results Table” as a table in CSV file format. The PDF plots of the results shown can be copied and saved on a local machine.

**Fig 2:**
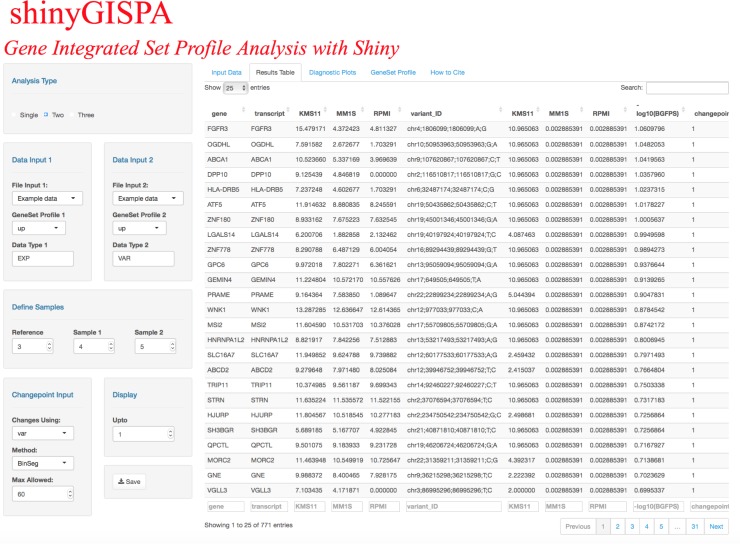
Results table snapshot of the shinyGISPA user-interface with the computed profile statistic scores using the example data sets.

**Fig 3:**
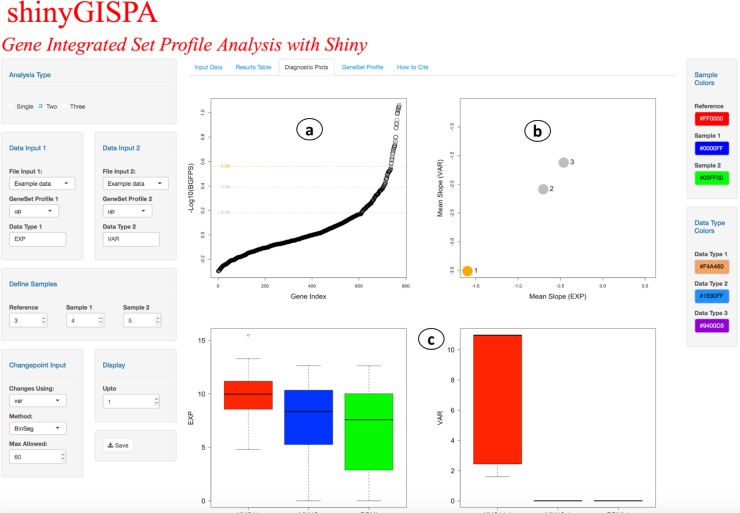
Results diagnostics plots snapshot of the shinyGISPA user-interface using the example data sets.

**Fig 4:**
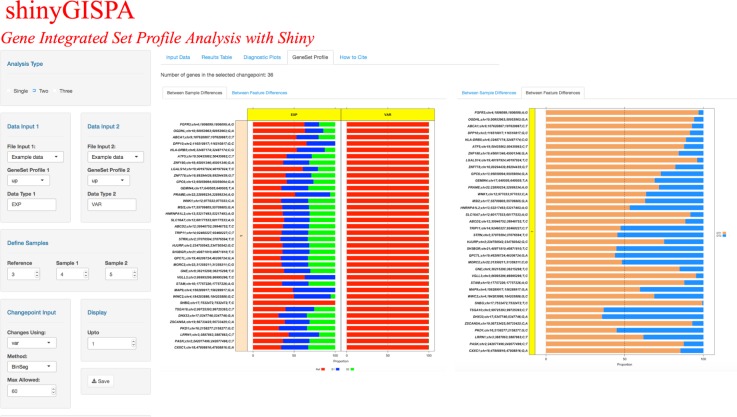
Sections of shinyGISPA showing gene sets profile support by “between sample differences” and “between feature differences” using the example data sets.

The results are output in four separate tabs. (1) *Input Data*. Summarizes the user input data in terms of the input number of genes (or rows), number of samples (or columns), user-defined reference sample, and a box plot of the data distribution and table view of the input data set. The user can set the color palette of choice with the “Sample Colors” side panel on the right to represent the three sample groups. (2) *Results Table*. Outputs the table of gene sets sorted by their profile statistic scores, which are then grouped according to identified change points for the user-selected profile. The profile statistics scores for each gene are computed using the GISPA method (please see method details in [3]). Figs 2–4 shows the results of a two-feature analysis run using the provided example data sets to identify genes that show support for the profile of increased expression and increased variant allele frequencies in the reference sample. The results table (Fig 2) can be searched, sorted, and filtered by any of the columns. (3) *Diagnostic Plots*. shinyGISPA generates, (a) *Change point plot*, an ordered plot of smallest (least desirable) to largest (most desirable) transformed profile statistic computed between features for each gene (circle), with identified breakpoints cutting the data to define segments or sets of genes that vary in their support for the molecular profile of interest. The topmost change point ‘1’ serves as the highest point in the plot line, indicated with an orange line, such that the genes above it show the most support for the profile in characterizing the reference sample versus the other samples. In general, the higher the change point, the less support for the profile of interest. (b) *Slope plot* helps in determining the selection of the number of change points, and hence the number of gene sets that show support for the user-defined profile. A slope defined by the ratio of the differences between the reference and each comparison sample within each data type for each gene is calculated and then averaged among the genes defined by each change point. The averaged slopes for each change point are plotted as circles, with the topmost, “best” profile (change point 1) shown in orange. Gene sets that satisfy a profile of increased change (e.g., increase gene expression with increase variant allele change) will have ‘small’ slope values that are depicted at the lower-left corner. Alternatively, gene sets that satisfy a profile of decreased change (e.g. decrease gene expression with decrease variant change) will have ‘large’ slope values that are depicted at the upper-right corner. The circle that is farther away from the other circles and closer to the respective corners of the plot indicate gene sets (i.e. change points) that show the topmost support for the profile of interest, though they vary in their degree of support. In Fig 3, genes selected based on the first change point only are shown as supporting the profile. (c) *Boxplots* representing the input data distribution of gene sets for the selected change points (under “Display” input panel) by sample groups for each data type. These plots allow the user to further assess the support for the profile based on the selected set of genes. (4) *Gene Set Profile*. The stacked bar plots in Fig 4 enables the users to visualize the breakdown among data types (‘between-feature differences’) that define a profile within each selected gene and the breakdown among the samples (‘between-sample differences’) within each data type. The former breakdown may be used to further identify prominent, driving features for a profile while the latter breakdown provides further confirmation of why the genes were selected as supporting the user-specified profile. The “Between Sample Differences” bar plot represents the differences among the samples, i.e., the percent contribution from each sample to the summed total of all samples by each data type. In Fig 4, the top most gene *FGFR3* is shown with increased expression (60%) in the reference sample (red-filled bar) compared to sample 1 (blue-filled bar) and sample 2 (green-filled bar), while *FGFR3* variant allele frequency is shown as only present in the reference sample (100%). The “Between-Feature Differences” plot represents the percent contribution from each feature (data type) for the profile by gene. Here, in the case of *FGFR3*, expression is shown as the most prominent feature, accounting for around 95% of the profile statistics as compared to the changes in variant that account for 5%. For these plots, the user can adjust the plotting parameters including the data type color palettes through the side menu panels.

## Results

### Validation

We have developed an open-access interactive web-based application for the GISPA method published in Kowalski et al., 2016. The method was validated for the characterization of the human multiple myeloma (MM) cell lines [3]. In specific, GISPA was able to identify both known and novel molecular profiles of MM cell lines within the context of a single- and two-feature analysis (see Table 1).

**Table 1:** GISPA identified known biomarkers of cell lines in MM.

MM Cell Line	IgH Tx	*FGFR3*	*TP53*	*CDKN2A*	*CDKN2B*	*KRAS*
KMS-11	t(4;14)	Y373C; Expressed	HD	WT	WT	WT
MM.1S	t(14;16)	WT	WT	HD	HD	G12A
RPMI_8226	t(14;16)	WT	E285K	WT	WT	G12A

*HD = Homozygous Deletion; WT = Wild type

### Applications

Our shinyGISPA tool provides the following flexibility to users in terms of performing an integrated data merging analysis: (1) any sample source, whether cell line or patient, is applicable, (2) data from different platforms with different scales can be input and combined together, (3) genome-wide or a selected subset of data can be used as input, (4) as few as a single sample per phenotypic group is applicable, and (5) one, two, or three-features of data in any combination can be used to define a molecular profile. Here we demonstrated the utility of shinyGISPA to a multiple myeloma data set; however, the tool is applicable to diverse cancer types or any other disease phenotype. One of the practical applications of our shinyGISPA tool is to scale up an experiment from cell lines to patient data. For example, it is typically the case that a researcher will first perform sequencing on cell lines to define which molecular changes characterize say resistant versus sensitive cell lines. For this purpose, there exists several public domain data on cancer cell lines of many genome-wide data types that a user may obtain to apply shinyGISPA. Once defined, the molecular alterations could then be examined in greater detail (and depth of coverage) using more focused NGS technologies, such as targeted sequencing, in tumors from patients. There are additional shinyGISPA applications such as in resolving subtype differences (e.g., glioblastoma) from use of different technologies to measure the same quantity. A premier example of this application is to identify genes with increased expression from one technology that also show increased expression of genes from another technology. Often times one struggles with using RNA-seq derived gene expression data for subtype identification an array-based gene expression derived signature, since the resulting subtypes do not show clear differentiation among samples as with the array data. In this case, one may use shinyGISPA to define a set of core genes from the signature set with increased expression in both the RNA-seq derived and the array-based data, specific to each subtype. In the same manner, our tool can be creatively used to filter and select gene array probes with consistent expression patterns to RNA-Seq gene transcripts. Additional, similar applications of shinyGISPA include its use to assess relationships between tissue-specific protein expression and phosphorylation. This tool can be extended to identify cell lines that are resistant (or sensitive) to chemotherapeutic drugs using IC50 values, as any quantitative measure may be applied other than molecular data. These are just a few of the several examples that highlight the tool’s capability of addressing a wide scope of molecular research area and in potentially going beyond a pure molecular analysis.

### Limitations

We have a dedicated private server to maintain this web application over the next five years after which we intend to upgrade considering the usage and incoming traffic. As opposed to requiring the complicated installations of several R packages and system libraries for the tool to work, shinyGISPA is a user-friendly web-based application that requires only good internet connectivity. The speed at which the results and visuals are generated will depend on the internet connection speed. It may take from a few seconds to a few minutes for analysis running in the background to finish depending on the size of the input data. When dealing with large data sets, or combining large genome-wide data sets as in two or three-feature analysis, it may take longer. In that case, the tool will display a progress bar notifying the users once the process is completed. For instance, with a data set size of 15,000 genes, the tool is able to generate results in a matter of 5 seconds. With data set size of 450,000 rows, results are generated in 2 minutes or less. With data sets of gene size 15,000, and probe size 450,000 in a two-feature analysis, it takes less than a minute to finish. For very large data sets that exceed the maximum upload size limit (>500MB) of the shiny server or unable to run on the browser, R source code from GitHub is made available and can be run locally from the command line, though processing speed is dependent on the local machine. The tool will not work if the input data files are not in the correct format. The tool will not generate any output if no change points are identified in the input data. Different output may be generated depending on the type of change and the version of change point R package used. At present, the maximum number of data types allowed in any combination is up to three. Among future implementations, shinyGISPA is scalable to incorporate more than three samples groups or more than three data types.

## Discussion

Identification of driver genes in cancer biology is crucial. It is even more important to incorporate changes from all molecular levels of high-dimensional data to proper understanding of multiple factors driving the tumor growth. This helps improve efficiency of predicting oncogenes or tumor suppressor genes associated with pathogenesis compared to genes based on individual data sets (e.g., gene expression profiles). Moreover, multiple molecular targets are needed to improve drug efficacy against cancer cells by targeting multiple molecular mechanisms, for instance, DNA methylation, an epigenetic mechanism often modify the function of the genes by regulating gene expression. Likewise, expression patterns vary with gene mutations and/or copy-number changes that may be linked to the clinical phenotype.

Our shinyGISPA tool provides biologists with easy access for integrating multiple genomic data types to define genes that support an *a priori* specified molecular profile characteristic of a phenotype. Additionally, the tool provides shinyGISPA insights about which data type is informative in characterizing each gene among a set of genes, thus hinting at specific molecular mechanisms that may play a more prominent role in the phenotype. For example, an increased expression of *FGFR3* is identified to be associated with a specific *FGFR3* Y373C mutation in KMS-11 (Fig 4). The ‘Between-Sample Differences’ panel in the ‘Gene Set Profile’ shows the stacked bar plot for each gene in different change points by sample class and data type. This function will be helpful for biologists to quickly evaluate an intriguing gene based on its similarity between genes profiles, or to validate their lab findings as proof of principle of the tool. Likewise, the ‘Between-Feature Differences’ panel will aid researchers in determining when and where each gene is contributing to a biological mechanism (e.g., expression appears as a prominent driving feature in *FGFR3* as compared to the variant allele frequency). The ‘Diagnostic Plots’ panel provides visual summary to assess whether the gene set selected in the topmost change point ‘1’ match the desired gene set profile in the sample class of interest when compared to the raw input data summary. The tool provides flexibility to users in choosing the change point cut-off, provided that more than one gene set meets the profile of interest. The most unique feature of this tool is the ability to specify a molecular change under ‘Gene Set Profile’ by data type, thereby allowing users to try all possible combinations of interactions among genes in a single setting.

The shinyGISPA tool is a simple, user-friendly interface that allows researchers to identify candidate driver genes within the context of similar, a *priori* specified molecule change that is characteristics of the disease phenotype of interest. shinyGISPA can combine and compare multiple levels of genomic to proteomic data, for instance mRNA or miRNA expression from transcriptome and microarrays, mutations from whole-genome or whole-exome sequencing, copy-number changes from whole-genome sequencing or SNP arrays, epigenetic changes from DNA methylation arrays or whole-genome bisulfite sequencing data, and proteomic data from mass spectrometry to name a few.

Although methods have been developed that associate gene sets with a phenotype of interest [2], they fail to address some commonly suffered problems. For instance, the most popular gene set analysis method (e.g., GSEA [7, 8]), is not able to identify distinguished gene sets relative to all other genes in a genome-wide data set, and is restricted to two sample groups that require greater than a single sample per group. Additionally, GSEA is restricted to examining differences between two sample groups with respect to changes in a single, gene expression data type. Furthermore, in comparison to GISPA, GSEA-derived gene sets are characterized by the activity levels of curated biological pathways rather than the molecular changes of individual genes or set of genes. The shinyGISPA tool is the only currently available interface that is based on a data merging approach to combine heterogeneous molecular data types with the goal of defining gene sets that support a specified molecular profile of change using as few as a single sample relative to other single samples.
